# Supplementary material for: Stable Isotope Ratio Analysis for the Authentication of Natural Antioxidant Curcuminoids from Curcuma longa (Turmeric)
Source: Antioxidants (Basel). 2023 Feb 16;12(2):498. doi: 10.3390/antiox12020498 (PMC9952763; doi:10.3390/antiox12020498)
Supplement: Supplementary file 1 [file antioxidants-12-00498-s001.zip › antioxidants-2200914-supplementary.pdf]

Table S1. δ^2H and δ^{13}C stable isotopic composition and ^{14}C content of curcuminoids adulterated with different % of synthetic curcuminoids.

% of synthetic added	Carbon 14 %	δ^2H (‰, vs V-SMOW)	δ^{13}C (‰, vs V-PDB)
70	25	19	-27.2
72	29	14	-27.8
65	33	8	-28.0
28	70.0	-52	-29.9
30	74	-53	-29.9
26	75.1	-67	-29.9
18	82.8	-67	-29.9
